# A full rat-scale model of the basal ganglia and thalamocortical network to reproduce Parkinsonian tremor

**DOI:** 10.1186/1471-2202-16-S1-P64

**Published:** 2015-12-18

**Authors:** Jan Moren, Jun Igarashi, Junichiro Yoshimoto, Kenji Doya

**Affiliations:** 1 Neural Computation Unit, Okinawa institute of Science and Technology Graduate University, Okinawa, Japan

## 

We aim to investigate the precise mechanisms underlying Parkinsonian symptoms by large-scale simulation of realistic neural network models including the basal ganglia (BG), thalamus, the motor cortex and spinal cord.

Parkinson 's disease is a mid- or late-age degenerative disorder of the central nervous system. The symptoms include akinesia, resting tremor, rigidity and gait problems. A principal cause of PD is the loss of dopaminergic neurons in the substantia nigra pars compacta. How the loss of dopamine transmission causes the symptoms remains unclear.

Although it was previously assumed that the tremor is caused by enhanced striatum->GPe (external Globus Pallidus) inhibition, recent experimental data suggests that enhanced GPe->STN (Subthalamic nucleus) inhibition generates synchronized STN rebound bursts to initiate tremor [1]. While the BG oscillation is in beta-band (8-15Hz), the final physical tremor is 4-8Hz, half the BG frequency. The oscillation thus undergoes sub-harmonic responses downstream of the BG, likely in the thalamocortical network.

As the oscillation originates through intercellular interactions and depends on multiple distinct areas, we connect multiple spatially-organized network models to form an integrated simulation system. We construct separate BG and thalamocortical models using NEST and connect them with MUSIC. The separate models enable concurrent development by different researchers, and the clean separation simplifies the integration work.

The BG model is based on Shouno et al. [2,3]. All subareas are modeled at the full spatial size and neuron numbers of the rat. The striatum includes medium-spiny D1 and D2 neurons and fast-spiking interneurons, with D1 neurons projecting directly to the GPi (internal Globus Pallidus) and D2 neurons to the GPe-STN indirect pathway. The total number of neurons exceeds 3 million. The neuron models are conductance-based with static or STDP synapses.

The thalamocortical network model consists of four neuron types in the thalamus and five layers of the primary motor cortex, with neuron numbers, spatial layout and connections based on experimental data. The cortical surface size is 3mm^2 ^and consists of ~180K neurons organized in a stochastic 3-d configuration using Integrate-and-Fire neurons.

We show the BG in a normal and a PD-like state with a ~14Hz oscillation in the GPi arising from STN-GPe interaction. Inhibitory GPi-thalamus connection may evoke rebound oscillation in the thalamus, which causes oscillatory output from the cortical pyramidal tract neurons.
